# Elements of the Theory and Practice of Medicine

**Published:** 1829-01-01

**Authors:** 


### ELEMENTS OF THE THEORY AND PRACTICE OF PHYSIC, DESIGNED FOR THE USE OF STUDENTS. By *George Gregory*, M.D. &c. Third Edition, pp. 696.

THE performance which now lies before us, in reaching its *third* edition, has abundantly substantiated the character we gave of it, and since, from its very favourable reception in America as well as here, it promises to become the medical text-book of our day, we have been induced to notice the appearance of this new impression, and to make our readers acquainted with the *additional* claims it has upon their notice.

We have been watching for some time the efforts which *some* of our cotemporaries are making to squib into notoriety, by puffs and eulogies, the most drivelling effusions of undigested thought, and to sink, by low abuse and unfair quotation, works of genius, got up with labour, which scorn to cater to prejudice, or to base their popularity upon the merits of a title-page. If ballad mongers and pamphleteers *will* step out of their legitimate department, and assume the censorship of medicine, let them have the *industry to read* what they have the *hardihood to criticise;* for, although neither the public nor the profession expect from them what must emanate from talent, they require honesty at their hand; and he who comes forward with professions to cure, should not, if he failed in the attempt, murder the patient he volunteered to save. Such advertisers are the bane of science;—the false-lights of society. Indolence and industry, sense and stupidity, truth and trash, meet with the same reception, and are either lauded without rule, or condemned without reason. Thus, do the deserving receive injustice, and the presumptuous encouragement: the public are deceived; truth can make no progress for the prevalence of error; and the interests of medicine are sacrificed at the shrine of some petty prejudice, or some avaricious principle.

The general outline of these Elements remains unaltered as to arrangement, and their general principles remain unchanged; but the interval, which has elapsed since the publication of the former impression until the present, has furnished the Doctor with several opportunities of enhancing their value. An edition of this work was published at Philadelphia, with very copious notes and additions by Drs. Potter and Colhoun; the contributions of these writers have been occasionally adopted, and, independent of a careful revision of the whole, and the addition of the author's recent experience, several topics unnoticed in the preceding editions have been introduced; as delirium tremens, paralysis agitans, cachexia africana, hepatalgia, and erythema nodosum. In the few following observations we shall confine ourselves to a few points, and, referring to our general analysis as formerly given, allude to such portions and subjects as are especially interesting.

In regulating our treatment of fever, several points of very great importance are insisted on;—the first, in their tendency to run a certain course, if neglected at their commencement; in the second, he draws an argument of very great force, to induce us to have recourse to early measures, from the disposition which exists in all febrile diseases to local congestion and inflammation, which so frequently terminate in the entire destruction of the organs implicated;—in the third, he proves that every symptom should be cautiously attended to in our treatment; and, lastly, that the nature of the epidemic should be consulted. No advice of greater importance can be given than what is contained in the last two particulars; and ignorance or neglect of them has been the principal reason, why the contrariety of the remedies we are in the habit of employing has created much scepticism as to their efficacy, and why the mortality occasioned by this disease has been so appalling during some epidemics. Routine practice is the bane of medicine;—a relic of ignorance which the enlightened condition of the present day ought to banish from our profession. Symptoms are the only safe-guides for the physician, as they are the only *language*

of disease; and he who learns not to study their every accent and variety of import, cannot advance one step along the path of science, and will destroy more life than his system can ever save. The etiology of all diseases is very obscure, but that of fevers is peculiarly so; and if we overlook the only lights which can aid our researches into the mysteries of their operation, our treatment, however ennobled by antiquity and enhanced by names, is resolvable into as pure empiricism, as is the conduct of him who gives digitalis wherever he recognizes dropsy, or tonics when his patient complains of weakness.

Speaking of the influence of soil in modifying fever, he alludes to a question of very great interest:—

"These *conditions of the soil* are not merely the occasion of *agues*, but they serve to modify the character of continued fever, and of any other febrile disease which may happen to occur in such a situation. This principle in pathology we have already had occasion to allude to, when treating of continued fever. A tendency is thus given to *exacerbation* and *remission* in the symptoms of the disease; and it is not improbable, that many cases of what might be considered genuine remittent fever from marsh exhalations, are, in fact, cases of common continued fever from cold, modified by peculiarities of soil. This consideration involves the question of the relation in which intermittent and continued fevers stand to each other; and before entering on the method of treatment in intermittents, I may shortly advert to it. It is contended by some, that all fevers are closely allied to each other, in their nature, in the operation of their exciting causes, and in their method of cure. Other pathologists again maintain, that intermittent and continued fevers are *essentially* different from each other, and that *essential* differences exist in the principles of their treatment. Our knowledge of the pathology of fever is hardly sufficient to authorize a *decided* opinion on the speculative question at issue, but it is certainly better for the student to view them as *distinct* classes of disorders." 77.

The author has added to his former list of *phlegmasial* diseases *delirium tremens*, and, being an affection of great obscurity, we will subjoin an extract from his remarks upon it, as a specimen of the interesting novelties with which the present edition is enriched.

"It has for its pathognomonic symptoms, delirium (sometimes fierce, but more generally restrainable), delusions of sight, trembling of the hands or whole frame, and complete sleeplessness. Fever is here seldom strongly developed, and the pulse wants the character of true inflammation. A like combination of symptoms, with the addition of damp perspirations, sometimes occurs in the latter stages of fever, and I have witnessed it as a sequel or metastasis of acute rheumatism. Under all circumstances, delirium tremens indicates extreme danger. It arises in a very large proportion of cases from the excessive use of ardent spirits; but a few instances have been traced to other sources, such as the poison of lead, the habitual use of opium, and strong mental emotion. It appears to have for its proximate cause a peculiarly excited state of the nervous system; but the occurrence of such symptoms in cases of extreme inanition would lead to the belief that *exhaustion of nervous power* expresses perhaps more accurately its intimate nature. Delirium tremens usually runs its course in about four or five days. It sometimes terminates in a fatal epileptic fit. It is universally admitted that this complaint does not admit of depletion by blood-letting. Much mischief indeed has followed its employment. Leeches however are occasionally useful, and sometimes in its early stage indispensable. The principal aim of the physician should be to calm and support the nervous system, and if possible to procure sleep. Opium answers all these indications, and must be given in full and frequently repeated doses. Where the complaint can be traced distinctly to the excessive use of ardent spirits, the accustomed stimulus must not be too rapidly withdrawn. Wine or brandy, in moderate quantities, should be administered. Æther, ammonia, camphor, and hyosciamus, have also been found beneficial. It is unnecessary to add, that moderate purging should also be directed, for in so disordered state of the nervous system, the secretions can scarcely fail to be greatly vitiated." 176.

In no department of our profession are the triumphs of modern discovery so conspicuous as in diseases of the chest, and in none can the friend of pathological research adduce more triumphant illustrations of the achievments of his science.

We need not go farther back for confirmation of this remark, than to the days of Baillie, in whose *Morbid Anatomy* will be found the sum and substance of all that was then known, with any degree of certainty, at least, upon this subject; yet, how meagre is the information it contains when compared with the writings of later authors! To pass by every other, the productions of Laennec alone have changed the face of medicine,—have imparted to it a tangibility which it had not hitherto possessed,—have poured the light of magic upon the science of diagnosis,—have rendered palpable to the testimony of sense, what had been hitherto subjects of conjecture, and have enabled the practitioner to link causes with their effects, external symptoms with internal conditions of disease,—to prescribe with an object, and to prognosticate with a reason. We can now trace the progress of consûmption, from the miliary tubercle to the suppurating abscess, affixing to each phenomenon as it appears, its proper quantum of importance. Bronchitis may no longer be confounded with phthisis, nor hydrothorax with spasmodic asthma. Diseases of the heart are distinguishable from mere functional disorder, and the effects of a muddled brain, or of a pampered stomach, may not be confounded with the fruits of congenital mal-formation, or structural disease.

The functions which are carried on within the chest are of vital importance, and, although they cannot be materially disturbed without exhibiting very ostensible symptoms, their disturbance may arise from very different causes, some of which only are within our control. Thus, dyspnœa may appear as a symptom of general fever,—of inflammation of the bronchial lining,—of inflammation of the serous membrane of the thorax,—of deposition into the pulmonary parenchyma,—of preternatural secretion from the glands of the bronchia,—of mal-formation of the parietes of the chest,—of hydrothorax,—of aneurism of the aorta,—of organic disease of the heart,—of spasmodic action of the respiratory muscles,—of cerebral disease, and of chylopoietic disorder! Here, then, is one function disturbed by, at least, twelve very different causes, and one symptom exhibited as our leading guide to their discovery! How insufficient such a guide will prove, must be admitted by the most sanguine symptomatologist; and, although some aid may be derived from *auxiliary* phenomena, the diagnosis formed upon such doubtful data must be often conjectural, and, when correct, must ascribe its success more to the contingencies of chance, than to the certainties of science. When, therefore, there exists so much room for error, and when even the most skilled in the mysteries of disease acknowledge the many doubts and difficulties with which they are encompassed, we are sorry to find an author of Dr. Gregory's distinction, treating the discoveries of Laennec with so much indifference, and infusing into the minds of the rising generation a contempt for the study of the stethoscope. It is, certainly, proper to be jealous of every innovation, in a profession so fond of novelty as ours; to investigate patiently the merits of each proposal; and to adopt with reserve what may appear improbable or strange. But prudence differs from scepticism; and the same love of truth which guards us against error, encourages improvement. The probationary days of the stethoscope have now expired, and the merits of both mediate and immediate auscultation have been satisfactorily established; and, although it was at first wise to scrutinize with severity, an invention (especially of Gallic birth) putting forth so many claims, and to admit it with reluctance into the list of discoveries, upon which the character of our age, in after times, was to depend; to oppose it any longer is nothing short of persecution, and must tend to diminish the good which it is calculated to effect. Although Dr. Gregory has been the leader of the anti-stethoscope party, we can predict with confidence the approach of his recantation; and, knowing that his opposition has proceeded from the best of all principles,—hatred of error,—we will hail his desertion of the *old school* with the most enthusiastic welcome, confident that the mind, which takes the trouble to examine what it deems valuable to adopt, will ever be the warmest advocate of the claims it has discovered; and that the enemy, who has been the last to resign his sword in the defence of a cause he considers honourable, will be the foremost in espousing the party he opposed, when sufficient evidence is adduced to convince him they are right.

Deeming it unnecessary for the Dr. and improper for ourselves to dwell any longer upon a work, which has met with such extensive circulation, we again dismiss it with increased feelings of respect for the talent of its author, and with sanguine anticipations of the success it will yet receive. It is written in a plain and easy style, which is familiar without being vulgar, classical without being laboured; and, although a few occasional slips do still occur, they are of so trifling a nature as not materially to mar the general accuracy of the performance. Truth is evidently the constant object of investigation, and, during all the intricacies through which his undertaking leads him, he seeks her in every labyrinth, pursues her through every difficulty, and when the obscurity of his subject nearly veils her from his sight, his expressions become cautious, and, with the candour of an honest guide, who sees not his path before him, he warns his reader of his doubts, and points his attention to the clue that is most likely to conduct him with safety along his way. He neither wraps himself up in the mysteries of ancient, nor idolizes the discoveries of modern physic. Paracelsus is heard when he speaks the truth, and Hippocrates is impugned when Nature contradicts him. The effects of disease are traced to the circumstances which produced them, and pathology is studied as a light to symptoms.

